# Synthesis and Characterization of Sol–Gelled Barium Zirconate as Novel MTA Radiopacifiers

**DOI:** 10.3390/ma17123015

**Published:** 2024-06-19

**Authors:** Hsiu-Na Lin, May-Show Chen, Pei-Jung Chang, Yao-Chi Lee, Chin-Yi Chen, Yuh-Jing Chiou, Chung-Kwei Lin

**Affiliations:** 1 Research Center of Digital Oral Science and Technology, College of Oral Medicine, Taipei Medical University, Taipei 110, Taiwan; tiffanylin1214@gmail.com (H.-N.L.); maychen@tmu.edu.tw (M.-S.C.); peronchang@tmu.edu.tw (P.-J.C.).; chencyi@fcu.edu.tw (C.-Y.C.); 2 Department of Dentistry, Chang Gung Memorial Hospital, Taipei 105, Taiwan; 3 School of Dentistry, College of Oral Medicine, Taipei Medical University, Taipei 110, Taiwan; 4 Division of Prosthodontics, Department of Dentistry, Taipei Medical University Hospital, Taipei 110, Taiwan; 5 Graduate Institute of Manufacturing Technology, National Taipei University of Technology, Taipei 106, Taiwan; 6 Department of Chemical Engineering and Biotechnology, Tatung University, Taipei 104, Taiwan; cindy1998123@gmail.com; 7 Department of Materials Science and Engineering, Feng Chia University, Taichung 407, Taiwan; 8 School of Dental Technology, College of Oral Medicine, Taipei Medical University, Taipei 110, Taiwan

**Keywords:** barium zirconate, sol–gel, calcination, radiopacity, diametral tensile strength, setting time, mineral trioxide aggregates

## Abstract

Barium zirconate (BaZrO_3_, BZO), which exhibits superior mechanical, thermal, and chemical stability, has been widely used in many applications. In dentistry, BZO is used as a radiopacifier in mineral trioxide aggregates (MTAs) for endodontic filling applications. In the present study, BZO was prepared using the sol–gel process, followed by calcination at 700–1000 °C. The calcined BZO powders were investigated using X-ray diffraction and scanning electron microscopy. Thereafter, MTA-like cements with the addition of calcined BZO powder were evaluated to determine the optimal composition based on radiopacity, diametral tensile strength (DTS), and setting times. The experimental results showed that calcined BZO exhibited a majority BZO phase with minor zirconia crystals. The crystallinity, the percentage, and the average crystalline size of BZO increased with the increasing calcination temperature. The optimal MTA-like cement was obtained by adding 20% of the 700 °C-calcined BZO powder. The initial and final setting times were 25 and 32 min, respectively. They were significantly shorter than those (70 and 56 min, respectively) prepared with commercial BZO powder. It exhibited a radiopacity of 3.60 ± 0.22 mmAl and a DTS of 3.02 ± 0.18 MPa. After 28 days of simulated oral environment storage, the radiopacity and DTS decreased to 3.36 ± 0.53 mmAl and 2.84 ± 0.27 MPa, respectively. This suggests that 700 °C-calcined BZO powder has potential as a novel radiopacifier for MTAs.

## 1. Introduction

Perovskite-structure materials, whose name originates from the mineral perovskite, CaTiO_3_, have attracted much research and development interest. Typically, they possess a general formula, ABX_3_, in which A and B are cations and X is an anion. In an idealized cubic unit cell, A is the larger cation (similar to Ca^2+^) occupying the corner positions of the cubic formation, B is the smaller cation (similar to Ti^4+^) sitting in the center, and X (similar to O^2−^) is located at the face-centered positions. The solid ion-conducting perovskite-structure materials exhibit unique piezoelectric, pyroelectric, and photoelectric properties and have been widely used in many applications [1,2,3], including sensing [4,5,6], light-emitting diodes [7], photocatalysts [8], solar cells or fuel cells [9,10,11,12], etc.

Perovskite-structure materials are used in dentistry as radiopacifiers in mineral trioxide aggregates (MTAs) for endodontic filling applications [13]. For instance, perovskite materials such as CaZrO_3_ and BaZrO_3_, which possess superior characteristics, including high mechanical, thermal, and chemical stability, are used in the commercial products RetroMTA^®^ (BioMTA, Seoul, Republic of Korea) and Theracal^®^ (Bisco, Inc., Schaumburg, IL, USA), respectively. Oh et al. [14] used tricalcium silicate (Ca_3_SiO_5_) and calcium zirconate (CaZrO_3_) to synthesize a calcium zircon–silicate cement (ZC) and investigate its performance in root canal sealing. After 21 days, ZC showed less endotoxin leakage compared to the Endosequence BC sealer^®^ (EBC, Brasseler, Savannah, GA, USA). TheraCal^®^, however, is a resin-modified calcium silicate matrix with BaZrO_3_ as the radiopacifier. Camilleri [15] reported that a calcium phosphate phase was formed for Theracal^®^ after immersion in Hank’s balanced salt solution. In addition, the leaching of calcium ions was much lower in TheraCal^®^ than in Biodentine. Meanwhile, Camilleri [16] studied the hydration reaction of tricalcium silicate-based materials with a 20% zirconium oxide or barium zirconate radiopacifier, solidified by various solutions. BaZrO_3_ was found to enhance calcium hydroxide formation.

Both pristine and doped barium zirconate materials can be synthesized via various physical and chemical wet processes, such as conventional solid-state sintering [17,18], mechanical milling [17], spray pyrolysis [19], precipitation [20], and sol–gel synthesis [21,22]. Bach et al. studied stoichiometric mixtures of BaCO_3_ and ZrO_2_ without sintering aids and performed solid-phase synthesis at 1200 °C with minimal mass loss [18]. Manju et al. [22] synthesized BaZrO_3_ nanoparticles using the sol–gel self-combustion method through the complexation of citrate with cations in the gel. The dried gel underwent self-sustaining combustion and was further calcined at 1000 °C for 8 h to obtain the 69.5 nm BaZrO_3_ powder product. Babu et al. [23] used sol–gel self-combustion synthesis technology to explore the effect of the pH value. Research has found that when pH = 1, the gel structure is the most uniform and many sites of BaZrO_3_ can be obtained to form ceramics that are evenly dispersed, without agglomeration, and with the highest sintering density. Braham et al. [3] used the sol–gel method to synthesize the perovskite materials BaTiO_3_, BaZrO_3_, etc. Titanate materials have a crystallization temperature (900 °C for BaTiO_3_ and 600 °C for PbTiO_3_), while zirconate materials begin to crystallize at relatively higher temperatures (1000 °C for BaZrO_3_ and 680 °C for PbZrO_3_). All can obtain non-centrosymmetric perovskite structures and possess piezoelectric properties.

In the present study, barium titanate (BZO) powder was prepared using the sol–gel process, followed by calcination at 700–1000 °C. Commercial BZO (coded as C-BZO) was used for comparison. Commercial and calcined BZO powders were characterized and used as radiopacifiers for MTAs. The performance of MTA-like cements was investigated to determine the optimal parameters for potential endodontic applications.

## 2. Materials and Methods

### 2.1. Preparation and Characterization of Barium Zirconate

Barium acetate (Ba(CH_3_COO)_2_, purity 100%, Merck KGaA. Ltd., Darmstadt, Germany) and zirconium(IV) n-propoxide (Zr(OCH_2_CH_2_CH_3_)_4_, 70 wt.% in 1-propanol, Merck KGaA. Ltd., Germany) were used as the precursor reagents for the sol–gel process. The synthesis procedures were described as following: 5.1 g of barium acetate was dissolved in 20 mL glacial acetic acid (CH_3_COOH, purity 100%, Merck KGaA. Ltd., Germany) at 80 °C. An amount of 6.5 g of zirconium(IV) n-propoxide was dissolved in 20 mL n-propanol (purity 85%, Wako Pure Chemical Industries, Ltd., Tokyo, Japan) and added dropwise into barium acetate precursor solution. Deionized (DI) water (1 c.c.) was added into the solution, which was then aged for 6 h at 80 °C with magnetic stirring. After aging, the solution was dried at 80 °C for 1 day. The dried powder was then calcined at various temperatures (700, 800, 900, and 1000 °C) for 2 h. The resulting products were washed sequentially with 1 M formic acid, ethanol, and DI water. The final product was dried at 90 °C for further characterization. The sol–gelled and 700–1000 °C-calcined BZO powders were coded as BZO-7, -8, -9, and -10, respectively. In addition, commercial barium zirconate (coded as C-BZO, Sigma-Aldrich, St. Louis, MA, USA) was used for comparison.

A thermogravimetric analyzer (TGA-2, Mettler-Toledo, Greifensee, Switzerland) was used to examine the dried sol–gelled powder to determine the thermal properties. Thermogravimetry analysis (TGA), derivative thermogravimetry (DTG), and differential scanning calorimetry (DSC) were performed by heating from 25 to 900 °C, with a heating rate of 10 °C/min, under ambient atmosphere.

The crystalline structures of sol–gelled BZO powders calcined at various temperatures were investigated by the X-ray diffraction technique with an X-ray diffractometer (XRD, Bruker D2 PHASER, Billerica, MA, USA) using Cu Kα radiation (λ = 1.542 Å) that was operated at 30 kV and 10 mA. The experiment was carried out in the Bragg–Brentano geometry and the XRD patterns were recorded in the 20–80° 2θ range, using a 0.02° step size, a 0.8 s step time, and a 1.43°/min scan speed with a LynxEye detector. The XRD patterns were analyzed using Bruker’s proprietary software (Version 4.1.1, Bruker-AXS Diffrac EVA, Bruker, Madison, WI, USA). The Scherrer formula was used to estimate the average crystalline size [24]. In addition, field emission scanning electron microscopy (FE-SEM, Hitachi SU8000, Tokyo, Japan) was used to examine the powder morphologies of various sol–gelled calcined BZO powders. The particle sizes of various BZO powders were measured by outlining a region of each particle on SEM images to compute Feret’s diameter by the imaging processing software, Image J 1.39f (Wayne Rasband, National Institutes of Health, Bethesda, MD, USA). At least fifty measurements were performed to calculate the mean and standard deviation of each BZO powder [25].

### 2.2. Preparation and Evaluation of MTA-Like Cements

Portland cement (80 wt.%) and radiopacifier (20 wt.%; C-BZO or sol–gelled BZO powder) were mixed by a homogenizer (Prep-CB6, Medclub Scientific Co., Ltd., Taoyuan, Taiwan) for 10 min. The blended powder was added to deionized water using a powder-to-water ratio of 3 and mixed further by a Vortex-Genie 2 mixer (Scientific Industries, Inc., Bohemia, NY, USA) for 15 s. The so-obtained pastes were filled into an acrylic mold. The setting times of MTA-like cements was determined using a Vicat needle (Jin-Ching-Her Co., Ltd., Yunlin County, Taiwan) (300 g and 1 mm diameter) using rod-like (6 mm diameter and 5 mm height) samples. The initial setting time was determined when the depth of impression was less than 1 mm, whereas the final setting time was zero. MTA-like cement samples were placed in an incubator that was set at 37 °C and 100% relative humidity for one day or 28 days to simulate the oral environment.

In addition to the setting times, the performance of the MTA-like cements was evaluated according to its radiopacity and diametral tensile strength. The radiopacity was examined using a VX-65 dental X-ray system (Vatech Co., Yongin Si, Gyeonggi-Do, Republic of Korea). The X-ray was generated using a 62 kV voltage and a 10 mA current. Disc-shape (10 mm diameter and 1 mm thickness) samples and a referenced step-wedge aluminum block were exposed simultaneously for 0.64 s at 30 cm. A Kodak size 2 CR imaging plate (Eastman-Kodak Co., Rochester, NY, USA) was used to record the images, which were analyzed further with Image J software (version 1.54b, Wayne Rasband, National Institutes of Health, Bethesda, MD, USA). Diametral tensile strength (DTS) of rod-like (6 mm diameter and 5 mm height) samples was obtained using a texture analyzer (TA. XT plus, Stable Micro System, Godalming, UK) at 6.0 mm/min crosshead speed. DTS was calculated using the equation DTS = 2F/πbw, where F is the maximum applied load (N), b is the diameter (i.e., 6 mm), and w is the height (5 mm).

## 3. Results and Discussion

### 3.1. Preliminary Evaluation of Commercial Barium Ziconate Powder as Radiopacifier

**Figure 1:**
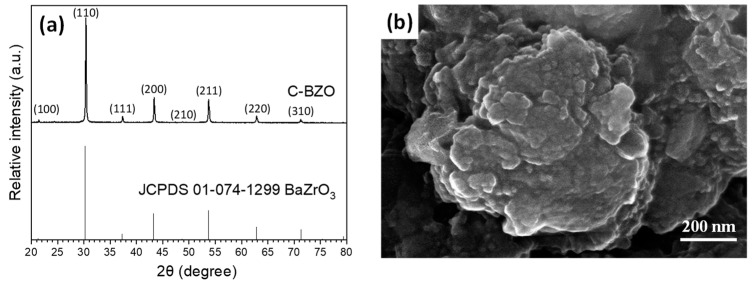
(**a**) XRD pattern and (**b**) SEM photo of commercial barium zirconate (C-BZO) powder.

Before preparing the sol–gelled barium zirconate (BaZrO_3_, abbreviated as BZO), commercially available BZO (coded as c-BZO) was used as a prototype and examined for radiopacifying application. Figure 1 shows the XRD pattern and SEM image of the C-BZO powder. It can be noted from Figure 1a that C-BZO exhibited a cubic perovskite crystalline structure, as referenced by JCPDS No. 01-074-1299, whereas the SEM image (Figure 1b) showed a severe agglomerated C-BZO powder. The morphology of C-BZO exhibited sintering phenomena, suggesting that it may have been prepared by a chemical method followed by calcination at a relatively high temperature or for a longer time.

**Figure 2:**
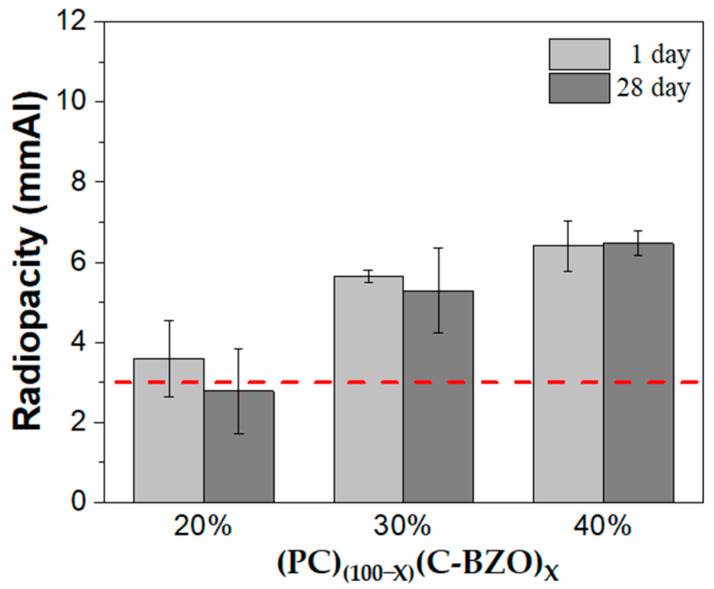
Radiopacity of MTA-like cements prepared by adding 20, 30, and 40% of commercial BZO powder. The red dashed line indicates the ISO standard requirement (3 mmAl).

The C-BZO powder was then used as a radiopacifier to prepare MTA-like cements for radiopacity and diametral tensile strength (DTS) evaluation, as shown in Figure 2. The radiopacity was 3.59 ± 0.96 mmAl for 20% C-BZO-added MTA-like cements. A decrease in radiopacity can be observed after storing in a simulated oral environment. After 28 days, it decreased to 2.78 ± 1.05 mmAl and did not satisfy the ISO standard requirement of 3 mmAl [26]. Since the radiopacity increased with the increasing amount of radiopacifier, it increased, respectively, to 5.65 ± 0.16 and 6.41 ± 0.63 mmAl with 30 and 40% C-BZO addition, as shown in Figure 2. Even after 28 days of simulation, their radiopacities were 5.29 ± 1.05 and 6.47 ± 0.31 mmAl, respectively. No significant differences in radiopacity can be observed after 28 days of storage.

**Figure 3:**
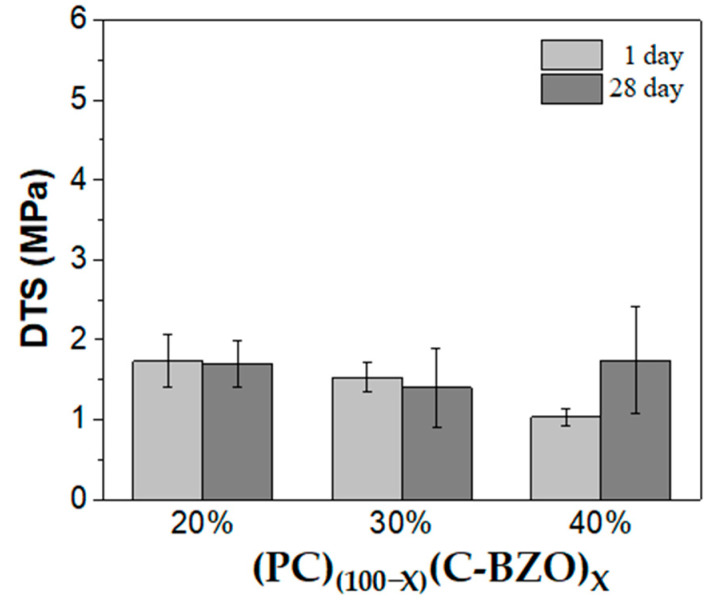
Diametral tensile strength of MTA-like cements prepared by adding 20, 30, and 40% of commercial BZO powder.

Figure 3 shows the DTS results corresponding to those shown in Figure 2. In contrast to the radiopacity performance, which increased with the increasing amount of C-BZO, the DTS exhibited a downward trend, measuring 1.73 ± 0.33, 1.53 ± 0.19, and 1.03 ± 0.11 MPa after 1 day with 20, 30, and 40 wt.% C-BZO addition, respectively. After 28 days of simulated storage, the DTS was 1.69 ± 0.29, 1.40 ± 0.49, and 1.74 ± 0.67 MPa, respectively. No significant variation can be observed for 20 and 30 wt.% C-BZO, whereas the MTA-like cements with 40% C-BZO added exhibited a relatively large increase after 28 days.

### 3.2. Synthesis and Characterization of Sol–Gelled BaZrO_3_

**Figure 4:**
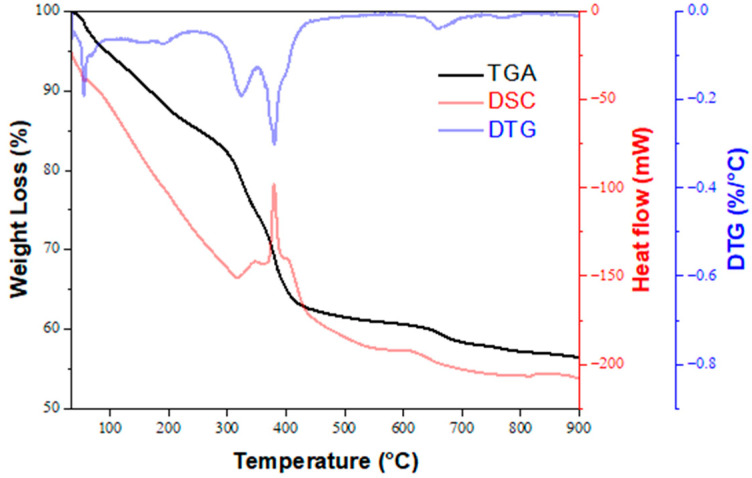
Thermogravimetric analysis (TGA), derivative thermogravimetry (DTG), and differential scanning calorimetry (DSC) curves for as-prepared sol–gelled BZO powder.

A preliminary evaluation using C-BZO powder revealed that most of the radiopacity satisfied the 3 mmAl requirement, while the DTS was smaller than 2 MPa. Further investigations using sol–gelled BZO powder were attempted. Figure 4 shows the thermal analysis results of the as-prepared sol–gelled BZO powder. As shown by the TGA curve in Figure 4 (black line), the weight loss can be briefly divided into three stages, according to the change of slopes: the first stage ranges from room temperature to 306 °C, the second stage from 306 °C to 418 °C, and the third stage from 418 °C to the end of the experiment. During the first stage, the weight loss of the sol–gelled powder was 19%, and it can be attributed to the evaporation or burnout of water, ethanol, and acetic acid, etc. Within the second stage, two relatively rapid decreases in weight were observed (more than two times the first stage speed), with a total weight loss of 19%. Within the 306 °C to 352 °C portion of the stage, the weight loss was attributed to the oxidizing combustion of the ethyoxyl, butoxy, etc., whereas the other incidence of weight loss during the 352 °C to 418 °C portion was ascribed to the formation of barium carbonate (BaCO_3_) and zirconia (ZrO_2_) [27]. These were confirmed by the corresponding exothermic peaks in the DTG (blue line) and the DSC curves (red line) at ~330 °C and 380 °C. At the last stage, a sluggish weight loss (~7%) was noticed from 418 °C to 900 °C, owing to the reaction between BaCO_3_ and ZrO_2_, which resulted in the formation of BaZrO_3_ and the release of CO_2_ gases.

**Figure 5:**
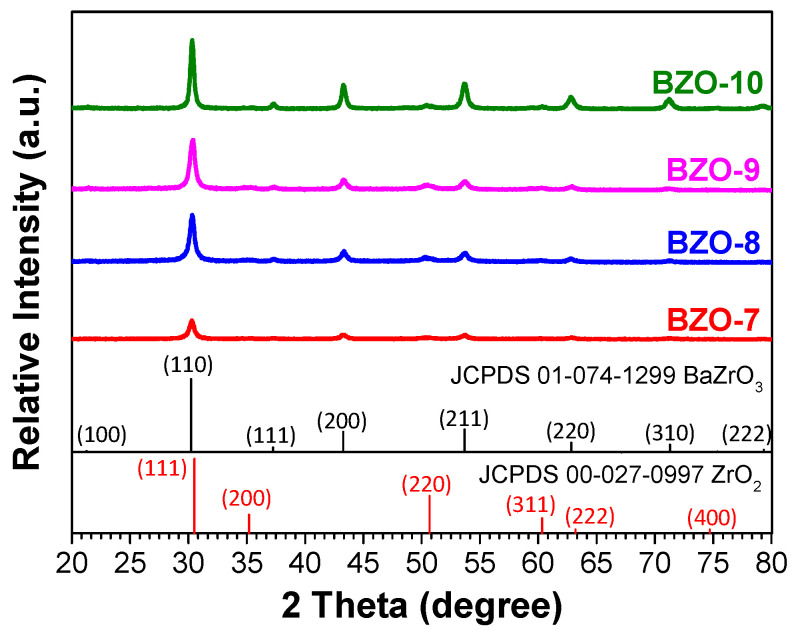
XRD patterns of sol–gelled BZO powders after calcination at 700, 800, 900, and 1000 °C for 2 h. The sol–gelled and 700–1000 °C-calcined BZO powders were coded as BZO-7, -8, -9, and -10, respectively.

**Figure 6:**
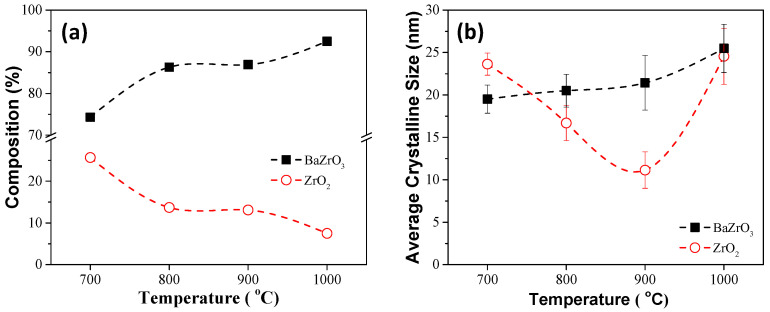
(**a**) Percentage of composition and (**b**) average crystalline size of sol–gelled BZO powders calcined at 700, 800, 900, and 1000 °C for 2 h.

**Figure 7:**
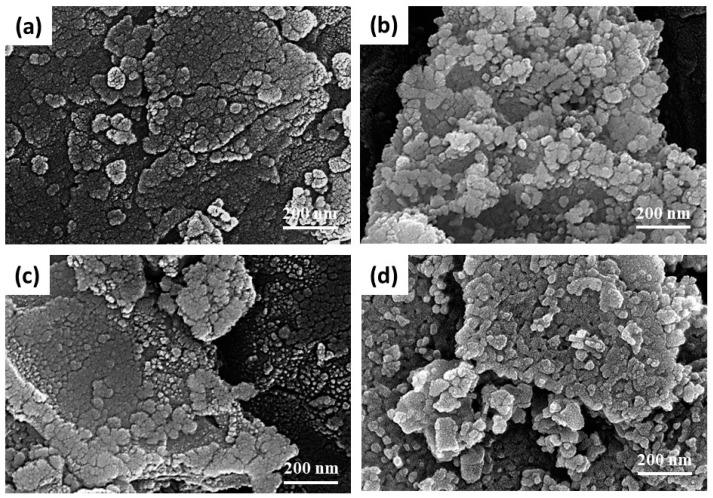
SEM images of sol–gelled barium titanate calcined at (**a**) 700, (**b**) 800, (**c**) 900, and (**d**) 1000 °C for 2 h.

**Figure 8:**
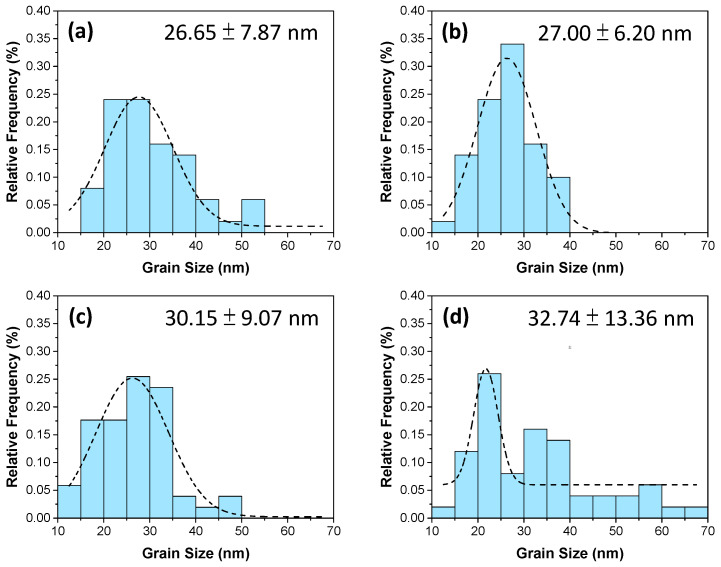
Histogram analysis of grain sizes from SEM images for sol–gelled barium titanate calcined at (**a**) 700, (**b**) 800, (**c**) 900, and (**d**) 1000 °C for 2 h.

Based on the thermal analysis results, the calcination temperature was set to 700–1000 °C for 2 h, respectively. Figure 5 shows the X-ray diffraction patterns of the calcined BZO powders, where the major BaZrO_3_ (JCPDS No. 01-074-1299, cubic $Pm\bar{3}m$ space group, a = 0.418 nm) and the minor ZrO_2_ (JCPDS No. 00-027-0997, cubic $Fm\bar{3}m$ space group, a = 0.509 nm) phases existed, and that the peak intensity increased with the increasing calcination temperature. The XRD results were analyzed further using the Rietveld fitting method [28,29]. The average crystalline size was calculated using the Scherrer formula [24]. Though standard measurement was not performed to determine the instrumental broadening of the X-ray diffractometer, a shape factor of 0.89 and an instrumental broadening of 0.05 (as suggested by the manufacturer) were used to estimate the average crystalline size and compared later with the results from SEM observation. Figure 6 shows the phase percentage and average crystalline size of BZO as a function of calcination temperature. It can be noted that the percentage of the BaZrO_3_ phase, shown in Figure 6a, increased with the increasing calcination temperature. It increased from 74.3% for BZO-7 to 92.5% for BZO-10, whereas the percentage of ZrO_2_ exhibited a reverse trend and was 25.7% and 7.5%, respectively. Figure 6b shows the average crystalline size of the corresponding BZO and ZrO_2_. The average crystalline size of BZO was 19.5 ± 1.7, 20.5 ± 1.9, 21.4 ± 3.2, and 25.5 ± 2.8 nm for the calcination temperatures of 700, 800, 900, and 1000 °C, respectively. The average crystalline size of the minor phase ZrO_2_, however, decreased from 23.6 ± 1.3 nm for BZO-7 to a minimum of 11.1 ± 2.1 nm for BZO-9, then increased to 24.6 ± 3.3 nm for BZO-10, as shown in Figure 6b. In summary, the crystallinity, the percentage, and the average crystalline size of BZO increased with the increasing calcination temperature. Figure 7 shows the powder morphology of the sol–gelled BZO powder, calcined at various temperatures. A severe agglomeration of the powder was observed, and no distinct difference could be seen. Figure S1 shows the EDS mapping of a typical calcined BZO powder, where the Ba, Zr, and O elements were randomly distributed. This suggested that the BZO powders were formed uniformly. The perceptible individual particles were equiaxed grains, used to determine the average grain size with Image J software 1.39f [25]. Figure 8 shows the corresponding histogram analysis results of grain sizes for various calcined BZO powders. The grain size was 26.65 ± 7.87 nm for BZO-7, and it increased continuously to 32.74 ± 13.36 nm for BZO-10. The grain sizes determined by the SEM images were slightly larger than those estimated by the Scherrer formula (Figure 6b), whereas a similar trend (i.e., the grain size increased with the increasing calcination temperature) was noticed.

### 3.3. Calcined BaZrO_3_ as Radiopacifier for MTAs

**Figure 9:**
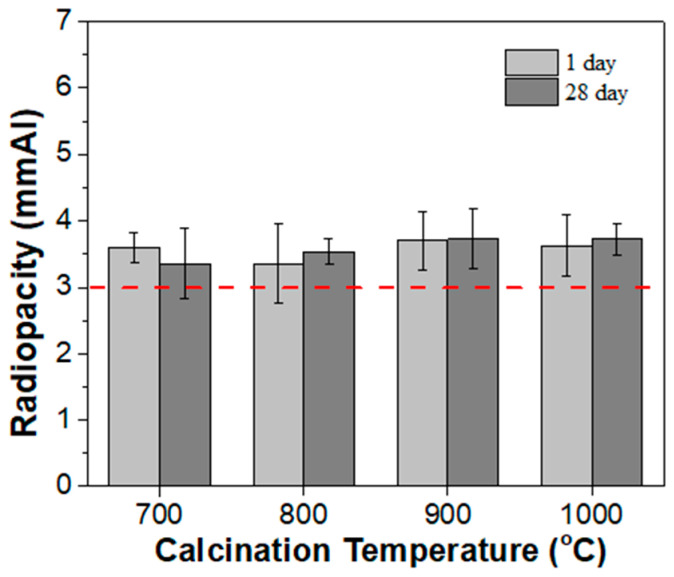
Radiopacity of MTA-like cements prepared by adding 20% of various BZO powders. The red dashed line indicates the ISO standard requirement (3 mmAl).

The calcined powders were used as radiopacifiers, mixed with Portland cement, mechanically milled, and then solidified with deionized water to prepare MTA-like cements. Figure 9 shows the radiopacity of MTA-like cements, where no significant differences in radiopacity can be observed using calcined-BTO powder. The radiopacity was 3.60 ± 0.22, 3.36 ± 0.59, 3.70 ± 0.44, and 3.63 ± 0.47 mmAl for BZO-7, -8, -9, and -10, respectively, which was similar to that of C-BZO (3.59 ± 0.96 mmAl, the one prepared by using commercial BZO powder, Figure 2). After storing in a simulated oral environment for 28 days, the radiopacities persisted and were 3.36 ± 0.53, 3.54 ± 0.20, 3.73 ± 0.46, and 3.73 ± 0.24 mmAl, respectively. No significant differences can be observed using BZO calcined at different temperatures and after 28 days of simulated storage. All of them satisfied the 3 mmAl requirement.

**Figure 10:**
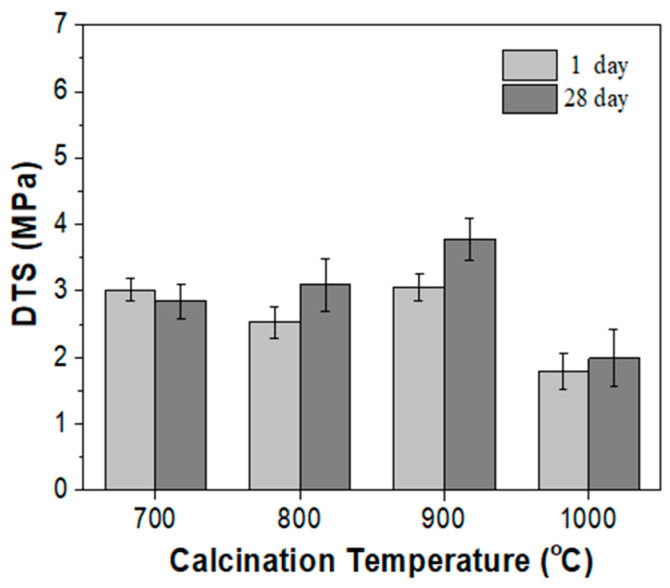
DTS of MTA-like cements prepared by adding 20% of various BZO powders.

A distinct improvement in DTS, however, was noticed for the BZO calcined at 700–900 °C. As shown in Figure 10, the DTS was 3.02 ± 0.18, 2.53 ± 0.23, 3.06 ± 0.21, and 1.79 ± 0.27 MPa for BZO-7, -8, -9, and -10, respectively. BZO-7, -8, and -9 exhibited a DTS higher than C-BZO (1.73 ± 0.33 MPa, Figure 3). Not only did BZO-10 possess a similar radiopacity, but it also had a similar DTS. After 28 days, the DTS was 2.84 ± 0.27, 3.09 ± 0.39, 3.78 ± 0.31, and 1.99 ± 0.43 MPa. For BZO-7 and BZO-10, there were no significant differences after 28 days of storage. An obvious improvement in DTS, however, was observed for BZO-8 and BZO-9. The improvement in DTS by using calcined BZO powder may be attributed to its relatively small crystalline size, compared to that of C-BZO powder. MTA-like cements prepared using calcined BZO powder exhibited better DTS values compared to those prepared with C-BZO (Figure 3, 1.73 ± 0.33 and 1.69 ± 0.29 MPa for 1 day and 28 days, respectively).

**Figure 11:**
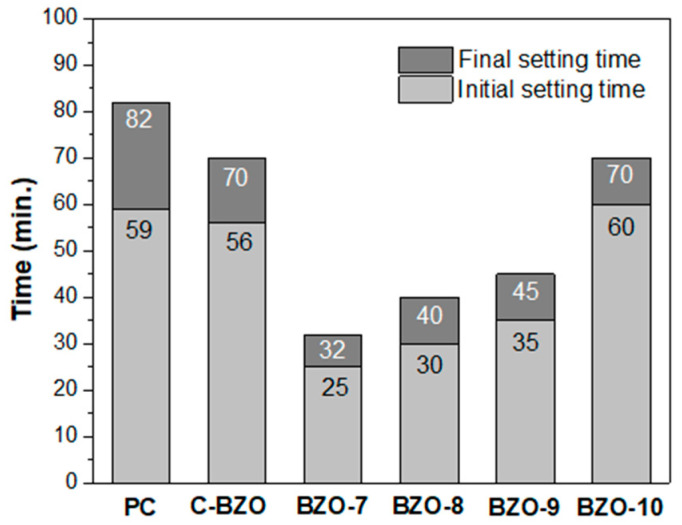
Initial and final setting times for selected MTA-like cements prepared by adding 20% of various BZO powders and solidified with powder/water = 3:1. Pure Portland cement (PC) was also used for comparison.

As demonstrated above, MTA-like cements prepared using calcined BZO powder exhibited similar radiopacity but better DTS compared to those prepared using C-BZO. The larger the amount of BZO addition in MTA-like cements, the higher the radiopacity becomes and the lower the DTS value decreases. It was also noted during the preparation of the MTA-like cements, the setting times were different when using C-BZO or calcined BZO powder. Since MTA-like cements prepared by adding 20 wt.% of calcined BTO powder exhibited a radiopacity that satisfied the 3 mmAl requirement, they were used to examine the setting times. Figure 11 shows the initial and final setting times for various MTA-like cements. It can be noted that the Portland cement exhibited the longest initial and final setting times of 59 and 82 min, respectively. The corresponding setting times were 56 and 70 min for C-BZO. For the calcined BZO powder prepared in the present work, the initial and final setting times increased with the increasing calcination temperature. They were 25 and 32 min for BZO-7 and increased gradually to 60 and 70 min for BZO-10, respectively. This suggests that the MTA-like cement prepared by adding 20% BZO-7 is optimal and has the potential to be used in practical applications.

## 4. Conclusions

BZO powder was prepared successfully via the sol–gel and calcination process. The higher the calcination temperature, the better the crystallinity became and the larger the average crystalline size of BZO grew. All MTA-like cements prepared by adding 20% BZO exhibited radiopacities larger than the 3 mmAl ISO requirement. In the present work, the MTA-like cement prepared by adding 20% BZO-7 powder exhibited the shortest initial and final setting times of 25 and 32 min, respectively, a radiopacity of 3.60 ± 0.22 mmAl, and a DTS of 3.02 ± 0.18 MPa. This suggests that the BZO-7 powder, possessing optimal properties, can potentially be used as a novel radiopacifier for mineral trioxide aggregates.

## Data Availability

No new data were created or analyzed in this study.

## Supplementary Materials

The following supporting information can be downloaded at: https://www.mdpi.com/article/10.3390/ma17123015/s1, Figure S1: (**a**) SEM image of 700 °C-calcined BZO powder, and EDX mapping of (**b**) Ba, (**c**) Zr, and (**d**) O elements for 700 °C-calcined BZO powder.
