# Supplementary material for: Synthesis and Characterization of Sol–Gelled Barium Zirconate as Novel MTA Radiopacifiers
Source: Materials (Basel). 2024 Jun 19;17(12):3015. doi: 10.3390/ma17123015 (PMC11205342; doi:10.3390/ma17123015)
Supplement: Supplementary file 1 [file materials-17-03015-s001.zip › materials-3058027-supplementary.docx]

Figure S1 shows the EDS mapping of a typical calcined BZO powder where Ba, Zr, and O elements were randomly distributed. This suggested that BZO powders were formed uniformly.


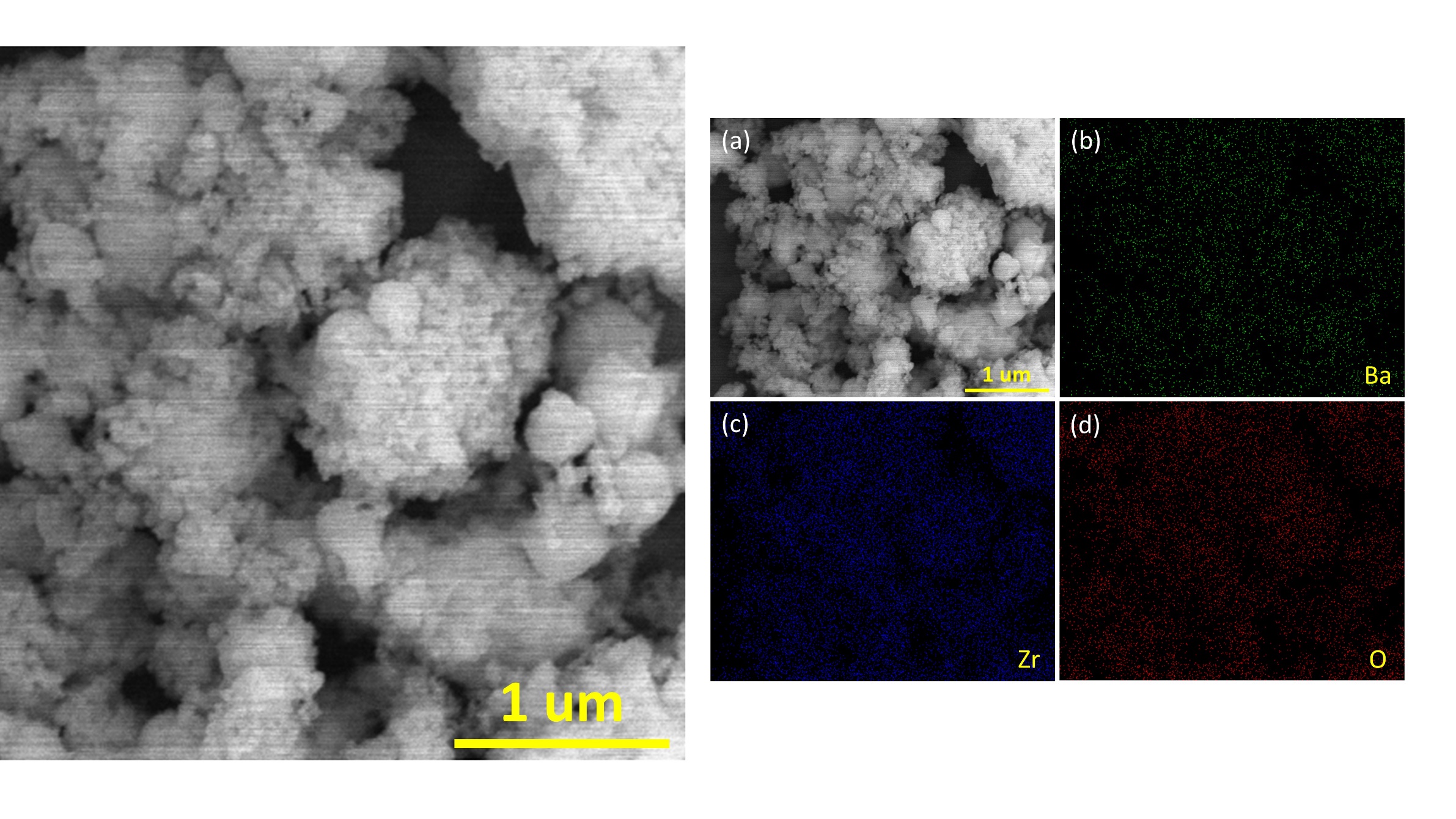


Figure S1: (a) SEM image of 700^o^C-calcined BZO powder, and EDX mapping of (b) Ba, (c) Zr, and (d) O elements for 700^o^C-calcined BZO powder.
